# Topical 7% Ectoine Modulates Cutaneous Response During Serial Vascular 1064-nm Nd:YAG Laser Therapy: A Randomized, Vehicle-Controlled Split-Face Study

**DOI:** 10.3390/biomedicines14092068

**Published:** 2026-09-15

**Authors:** Izabela Załęska, Urszula Goik, Anna Smuda

**Affiliations:** 1 Departament Cosmetology, Institute of Health, University of Applied Sciences Nowy Targ, 34-400 Nowy Targ, Poland; 2 Department of Engineering and Machinery for Food Industry, Faculty of Food Technology, University of Agriculture in Krakow, 30-149 Kraków, Poland; 3 International Association of Mesotherapy, 30-399 Kraków, Poland

**Keywords:** ectoine, Nd:YAG laser, skin erythema, skin barrier, transepidermal water loss, CIELAB, high-frequency ultrasonography, split-face study

## Abstract

**Background**: Repeated vascular laser exposure induces not only selective photothermal effects within blood vessels but also secondary responses in the surrounding skin. This study investigated whether topical 7% ectoine modifies the trajectory of cutaneous response during a series of 1064-nm Nd:YAG laser treatments. **Methods**: Thirty participants underwent four 1064-nm Nd:YAG laser treatments in a prospective, randomized, vehicle-controlled split-face study. Following each treatment, one side of the face received a formulation containing 7% ectoine (ECT), whereas the contralateral side received an identical vehicle without ectoine (vehicle control). Skin erythema, stratum corneum hydration, transepidermal water loss (TEWL), melanin index, and skin surface pH were assessed longitudinally. Redness was independently quantified using the CIELAB a* coordinate, while high-frequency ultrasonography was used to determine the proportion of low-echogenic pixels (LEPs). An untreated internal reference area (UIRA) was additionally assessed to characterize spontaneous temporal and environmental variability in skin parameters. The study was retrospectively registered in the ISRCTN registry (ISRCTN69645337). **Results**: Compared with the vehicle, ECT produced a significantly greater overall reduction in skin erythema (ΔΔ = −35.44 AU; 95% CI: −44.88 to −26.01; *p* < 0.001), accompanied by a greater reduction in CIELAB a* (ΔΔ = −1.18; 95% CI: −1.58 to −0.78; *p* < 0.001). ECT was also associated with a greater overall improvement in stratum corneum hydration assessed by corneometry (ΔΔ = +5.96 AU; *p* < 0.001), a greater reduction in TEWL (ΔΔ = −0.73 g/m^2^/h; *p* = 0.0012), and a greater decrease in LEPs (ΔΔ = −0.55 percentage points; *p* < 0.001). No significant between-treatment advantage was observed for the melanin index or skin surface pH. **Conclusions**: Topical application of 7% ectoine during serial vascular 1064-nm Nd:YAG laser therapy was associated with a distinct trajectory of cutaneous response compared with the vehicle alone. The effect was characterized by a greater reduction in the erythematous component, improvement in barrier-related parameters, and changes in dermal echogenic properties. These short-term instrumental findings support further investigation of ectoine as an adjunctive topical strategy during repeated photothermal procedures; their clinical significance requires confirmation using standardized clinical and patient-reported outcomes.

## 1. Introduction

Skin erythema is a visible manifestation of changes occurring within the superficial vascular bed and may represent either a physiological, transient response to a stimulus or a persistent or recurrent clinical condition. Its severity depends, among other factors, on blood vessel diameter and filling, cutaneous blood flow, and microvascular reactivity. In chronic or recurrent erythema, however, the presence of dilated vessels is not the only relevant factor. Increased skin susceptibility to triggering factors, including temperature changes, ultraviolet radiation, emotional stress, physical exertion, and irritants, may also contribute to the clinical presentation. Recurrent vascular responses may be further modulated by local inflammation, oxidative stress, and disturbances in epidermal barrier homeostasis [1,2,3].

The relationship between vascular and inflammatory components is bidirectional. Keratinocytes, immune cells, and endothelial cells participate in local intercellular communication through cytokines, chemokines, reactive oxygen species (ROS), and other mediators capable of influencing vascular tone and permeability. Activation of these pathways may promote vasodilation and increased cutaneous blood flow, thereby contributing to persistent clinically visible redness even after the initial stimulus has subsided. Skin erythema should therefore not be considered solely a consequence of visible vascular structures but rather a dynamic response involving interactions among the microcirculation, epidermal cells, and the local inflammatory environment [1,2,3].

One modality used to reduce the vascular component is the neodymium-doped yttrium aluminum garnet (Nd:YAG) laser emitting at a wavelength of 1064 nm. Its mechanism of action is based on selective photothermolysis, whereby absorption of laser energy by blood chromophores produces a controlled increase in intravascular temperature. Appropriate exposure parameters enable thermal injury to vascular structures while relatively limiting damage to the surrounding tissues. The 1064-nm wavelength is characterized by relatively deep penetration into the skin, allowing the Nd:YAG laser to target vascular structures located at different depths [4,5,6,7].

The biological effects of vascular laser therapy, however, are not restricted to the target chromophore. Energy transfer and the associated local increase in temperature also constitute a stress stimulus for surrounding skin cells. Laser exposure may therefore induce transient inflammatory and oxidative stress responses, release of cellular mediators, and alterations in microvascular reactivity. Clinically, these processes may manifest as post-treatment skin erythema, warmth, or transient edema. Post-laser redness may consequently comprise at least two overlapping components: the direct photothermal effect on vascular structures and a secondary, nonspecific erythematous-inflammatory response of the surrounding tissue. Qin et al. [8] demonstrated activation of inflammatory signaling in skin cells following 1064-nm Nd:YAG exposure, including the p38/MAPK and NF-κB pathways, whereas Said et al. [9] reported induction of HSP70 following non-ablative laser exposure, including Nd:YAG irradiation.

Distinguishing between these components is important when designing adjunctive skin care for vascular laser therapy. Such care should not attenuate the controlled thermal effect within the vessel, as this constitutes the therapeutic basis of Nd:YAG treatment. Instead, a potentially beneficial intervention should support the homeostasis of the surrounding skin and limit excessive or prolonged secondary stress responses without interfering with the intended photothermal effect [4,8,9].

This consideration may be particularly relevant when laser therapy is administered as a series of repeated treatments. The condition of the skin before each subsequent exposure reflects not only the effects of previous laser sessions but also the biological processes occurring during the intervals between treatments. If this period is characterized by persistent barrier dysfunction, increased vascular reactivity, or prolonged inflammatory signaling, subsequent procedures are performed on skin that is biologically different from its baseline state. Topical care applied between treatment sessions may therefore potentially influence not only post-procedural comfort but also the temporal trajectory of the cutaneous response throughout the entire treatment cycle [1,2,3,8].

From this perspective, ectoine is of particular interest. Ectoine is a naturally occurring compatible osmolyte produced by microorganisms adapted to highly stressful environmental conditions. Its biological activity should not be reduced solely to a moisturizing effect. Ectoine influences the organization of the aqueous environment surrounding macromolecules and biological membranes, thereby promoting their stabilization under conditions of dehydration, altered osmolarity, and temperature stress [10,11,12]. Studies of the stratum corneum have additionally demonstrated effects on keratin aggregate dispersion and on the kinetics of stratum corneum dehydration and rehydration [13]. These properties may be relevant to maintaining skin homeostasis during repeated exposure to physical stressors [10,11,12,13].

The potential relevance of ectoine to vascular laser therapy may, however, extend beyond its effects on water homeostasis and the epidermal barrier. Ectoine has been described as a cytoprotective compound that enhances the stability of cellular structures under stress conditions [10,11,12]. Experimental studies have demonstrated effects on cellular responses to thermal stress and mechanisms associated with heat-shock proteins [14]. Heat-shock proteins, including HSP70, function as molecular chaperones and contribute to protein protection and cellular homeostasis under conditions of elevated temperature [9,14]. In the context of procedures based on controlled photothermal effects, this mechanism provides a relevant rationale for investigating ectoine as a component of topical care between successive laser exposures [8,9,14].

A second potentially relevant mechanism involves modulation of the oxidative stress response. ROS are not merely products of cellular damage but also function as signaling molecules involved in the activation of inflammatory pathways. Excessive or prolonged ROS production may activate pathways regulating cytokine and chemokine expression and thereby alter the local inflammatory microenvironment [3,8]. Experimental studies have demonstrated protective effects of ectoine under oxidative stress conditions and its influence on cellular antioxidant systems [15]. Limitation of excessive ROS-dependent signaling may therefore represent one mechanism through which ectoine could modulate the cutaneous response to thermal stress [8,14,15].

Of particular relevance to skin erythema is the potential modulation of pro-inflammatory signaling. Keratinocytes are not passive components of the epidermal barrier; rather, they actively participate in cutaneous immune responses. In response to thermal or oxidative stress and tissue injury, keratinocytes may release cytokines and chemokines involved in communication with immune cells and microvascular structures. Pro-inflammatory mediators may indirectly increase vascular reactivity and endothelial permeability, thereby contributing to persistent redness and edema [1,2,3,8]. Experimental evidence indicating that ectoine can modulate inflammatory responses [14] therefore supports the hypothesis that it may also influence the nonspecific erythematous component developing after laser exposure.

Importantly, this hypothesis does not imply a direct vasoconstrictive effect of ectoine or selective action on blood vessels, as the currently available evidence does not support such conclusions. A more plausible mechanism involves indirect modulation of the microenvironment surrounding vascular structures through enhanced cellular resilience to thermal stress, attenuation of excessive oxidative stress, modulation of inflammatory signaling, and stabilization of epidermal barrier function [10,11,12,13,14,15]. Within this model, ectoine would not be expected to reduce the intended intravascular photothermal effect of the Nd:YAG laser but could potentially attenuate the secondary component of the cutaneous response contributing to prolonged or excessive redness.

The interaction between epidermal barrier function and inflammatory-vascular reactivity may also be relevant. Impairment of barrier integrity increases skin susceptibility to environmental stimuli and may promote keratinocyte activation and the release of pro-inflammatory mediators. Conversely, persistent inflammation may adversely affect normal barrier organization and function [1,2,3]. Supporting stratum corneum hydration and limiting excessive transepidermal water loss may therefore represent not only skin-conditioning effects but also potential components of reduced cutaneous reactivity between successive laser exposures [13,16].

The rationale for using ectoine in laser-exposed skin is further supported by previous studies. In a study evaluating topical care following ablative CO_2_ laser treatment, ectoine-containing formulations were associated with improved skin hydration, reduced transepidermal water loss, and lower skin erythema. The magnitude of the effect also depended on ectoine concentration and application frequency, with the greatest changes observed with regular application of the formulation containing the highest ectoine concentration. These findings suggested that the potential effects of ectoine following laser exposure may simultaneously involve barrier-related parameters and the erythematous response [17].

To date, evidence regarding the use of ectoine in laser procedures has been derived predominantly from ablative models [17]. It remains unclear whether comparable effects occur during non-ablative vascular laser therapy, in which blood chromophores constitute the primary target while the integrity of the epidermal surface remains preserved [4,5,6,7]. This model provides an opportunity to investigate the potential influence of ectoine not only on barrier function but also on the response of the surrounding skin to repeated thermal and inflammatory stress.

Furthermore, little is known about the effects of regular ectoine application throughout a complete series of repeated vascular laser treatments. In contrast to the assessment of a single exposure, a serial-treatment model enables evaluation of whether differences between ectoine and its vehicle develop progressively over the course of therapy and whether they persist after completion of the treatment series. Of particular interest is whether a potential reduction in the erythematous component occurs concomitantly with changes in epidermal barrier parameters and tissue properties, which could indicate broader modulation of the cutaneous response rather than a transient soothing effect alone.

Evaluation of such effects requires complementary methods capable of independently characterizing the erythematous component, epidermal barrier function, and tissue properties. Combining biophysical, reflectance-based, colorimetric, imaging, and ultrasonographic methods enables assessment of whether the potential effects of an intervention are restricted to a superficial clinical manifestation or extend to other levels of the cutaneous response [18,19,20,21,22,23,24,25,26,27,28,29,30].

**Figure 1:**
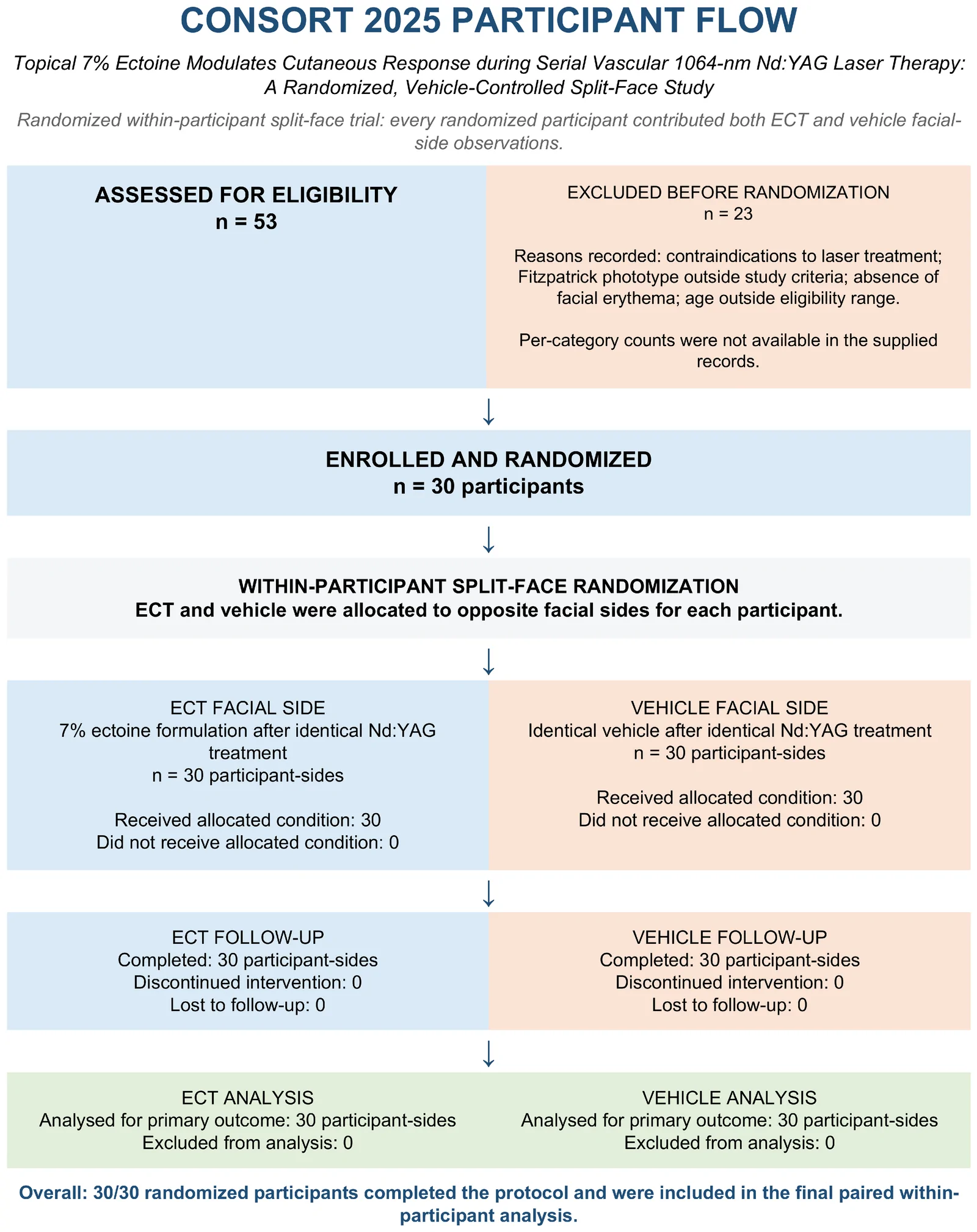
CONSORT 2025 Participate Flow.

A randomized split-face design additionally enables direct within-subject comparison of the two topical interventions. Both sides of the face are exposed to an identical Nd:YAG laser protocol and differ only in the topical formulation applied after treatment. This approach reduces the influence of interindividual variability in facial anatomy, vascular reactivity, skin phototype, barrier properties, and individual response to laser therapy. An untreated internal reference area (UIRA) was additionally included to characterize spontaneous temporal and shared environmental variability in skin parameters during the study period. The UIRA was not considered a third treatment arm and was not used for direct inferential comparisons of absolute values with the laser-treated facial areas because of anatomical and physiological differences between locations. The CONSORT flow is presented in Figure 1.

### 1.1. Study Aim

The primary aim of this study was to evaluate the effect of topical application of a formulation containing 7% ectoine on the temporal trajectory of cutaneous response during a series of four vascular 1064-nm Nd:YAG laser treatments using a randomized, vehicle-controlled split-face design.

Specifically, we investigated whether regular application of 7% ectoine between successive laser exposures modified the erythematous component of the cutaneous response and whether this effect was accompanied by changes in epidermal barrier function, stratum corneum hydration, skin surface pH, and ultrasonographic tissue properties. Sequential assessment time points enabled evaluation of both changes developing during the treatment series and responses persisting after completion of the full treatment protocol.

### 1.2. Study Hypothesis

We hypothesized that topical application of a formulation containing 7% ectoine following successive Nd:YAG laser treatments would modify the temporal trajectory of the cutaneous response compared with the identical vehicle without ectoine. Specifically, we expected a greater reduction in the erythematous component over the course of the protocol together with more favorable normalization of parameters related to epidermal barrier function between successive exposures.

We further hypothesized that the potential effect of ectoine would not result from direct vasoconstrictive activity or attenuation of the intended photothermal effect of the Nd:YAG laser within vascular structures. Instead, it was expected to reflect modulation of the surrounding tissue response to repeated thermal stress, potentially involving stabilization of cellular structures, modulation of oxidative and thermal stress responses, attenuation of excessive pro-inflammatory signaling, and support of epidermal barrier homeostasis.

## 2. Materials and Methods

### 2.1. Materials

The cosmetic formulations were prepared using the following ingredients: deionized water, ectoine (Alfa Sagittarius, Kraków, Poland), glycerin (Fagron, Modlniczka, Poland), panthenol (Sigma-Aldrich Chemie GmbH, Taufkirchen, Germany), sodium hyaluronate (1.0–1.5 MDa; Alfa Sagittarius, Kraków, Poland), gluconolactone and sodium benzoate (Arxada, Basel, Switzerland), sodium alginate (Agnex, Zaventem, Belgium), lactobionic acid (Cortex Chemicals Sp. z o.o., Tarnów, Poland), allantoin (Merck, Darmstadt, Germany), levulinic acid (Sigma-Aldrich Chemie GmbH, Taufkirchen, Germany), and carnosine (Synthetika Sp. z o.o., Łódź, Poland). Study design and timeline of the randomized split-face protocol, including ECT/vehicle allocation, the untreated internal reference area (UIRA), laser sessions, and T0–T2 assessments was presented in Table 1.

**Table 1:** Study design and timeline of the randomized split-face protocol, including ECT/vehicle allocation, the untreated internal reference area (UIRA), laser sessions, and T0–T2 assessments.

Etap	Laser/Intervention	ECT vs. Vehicle	Ocena
T0	Before first Nd:YAG session	Randomized split-face allocation; UIRA untreated	Baseline assessment
Tydzień 0	Nd:YAG session 1	First assigned application after treatment; then twice daily	—
Week 1	Nd:YAG session 2	Continue assigned formulations twice daily	—
T1/Week 2	Before session 3; session 3 then performed	Continue assigned formulations	Assessment before third treatment
Week 3 → T2	Session 4; T2 7 days later	Continue assigned formulations; UIRA untreated	Final assessment 7 days after session 4

### 2.2. Preparation of the Formulations

The cosmetic formulations used in the study were prepared from the raw materials listed in Table 2. First, an appropriate amount of allantoin was weighed using an analytical balance and added to approximately 60 g of deionized water. The mixture was heated to 40 °C and stirred using a magnetic stirrer at 500 rpm for 30 min. After dissolution of the allantoin, sodium alginate and sodium hyaluronate were added, and the formulation was subsequently mixed using a mechanical stirrer at 2000 rpm for approximately 3 h.

Glycerin, panthenol, and levulinic acid were then added and thoroughly mixed. In the remaining portion of deionized water, carnosine, lactobionic acid, and the preservative system consisting of gluconolactone and sodium benzoate were dissolved. This solution was combined with the first phase, and the complete formulation was mixed for approximately 30 min.

The resulting ectoine-free formulation constituted the vehicle control (vehicle). The active formulation (ECT) was prepared using the identical vehicle supplemented with 7% ectoine. The formulations were stored at 6 °C until use.

**Table 2:** Raw materials included in the recipes of tested formulations.

No.	Ingredients	Vehicle Without Ectoine (PLA) [g]	7% Ectoine Formulation (ECT) [g]
1	Ectoin	–	7.0
2	Glycerin	3.0	3.0
3	Panthenol	1.0	1.0
4	Sodium Hyaluronate 1.0–1.5 MDa	0.8	0.8
5	Gluconolactone and Sodium Benzoate	0.5	0.5
6	Sodium Alginate	0.4	0.4
7	Lactobionic Acid	0.3	0.3
8	Levulinic Acid	0.2	0.2
9	Allantoin	0.2	0.2
10	Carnosine	0.2	0.2
11	Aqua	q.s. to 100	q.s. to 100

### 2.3. Physicochemical Characterization of the Formulations

#### 2.3.1. pH Measurement

The pH of the prepared formulations was measured using an Elmetron CPC-505 pH meter. Measurements were performed in triplicate.

#### 2.3.2. Electrical Conductivity

The electrical conductivity of the cosmetic formulations was measured using an Elmetron CPC-505 conductivity meter. Measurements were performed in triplicate at 25 °C.

#### 2.3.3. Water Activity

Water activity was determined by placing a thin layer of each hydrogel formulation in a hermetically sealed measurement chamber and allowing the sample to equilibrate thermodynamically. Measurements were performed using an AQUALAB Series 4TE instrument, Heidolph Instruments GmbH & Co. KG, Schwabach, Germany. The instrument was calibrated at room temperature before analysis. Measurements were performed in triplicate at 25 °C.

#### 2.3.4. Rheological Measurements

The rheological properties of the formulations were evaluated using an RS-6000 rotational rheometer (Thermo Fisher Scientific, Bremen, Germany) equipped with a cone–plate geometry with a diameter of 35 mm, cone angle of 2°, and gap of 0.2 mm. Measurements were performed at 10, 20, and 32 °C, using an increasing shear rate ranging from 0.1 to 10 s^−1^.

### 2.4. Study Design

This study was designed as a prospective, randomized, vehicle-controlled split-face study to evaluate the effect of topical application of a formulation containing 7% ectoine (ECT) on the cutaneous response during a series of vascular treatments performed using a 1064-nm Nd:YAG laser.

In each participant, both sides of the face underwent an identical laser treatment protocol, whereas the topical post-treatment intervention differed between sides. One side of the face received the formulation containing 7% ectoine (ECT), while the contralateral side received the identical formulation without ectoine (vehicle). Thus, each participant served as their own control.

Allocation of ECT and vehicle to the right or left side of the face was individually randomized using a computer-generated randomization sequence. The formulations were provided in identical containers labeled with codes A and B. Participants and investigators performing the instrumental measurements remained blinded to formulation identity and side allocation until completion of data collection.

In addition to the randomized split-face comparison, an Untreated Internal Reference Area (UIRA) was designated on the forehead of each participant. This area was neither exposed to the Nd:YAG laser nor treated with either study formulation.

The UIRA was not considered a third study arm and, because of potential anatomical and physiological differences related to its location, was not used for direct comparison of absolute parameter values with the laser-treated facial areas. Instead, it served as a within-subject reference area for assessing spontaneous temporal variability and environmental variability shared across the study period.

The study consisted of four laser treatment sessions performed at 7-day intervals, accompanied by multiparametric assessment of the cutaneous response using biophysical, reflectance-based, imaging, and ultrasonographic methods.

### 2.5. Study Participants

Thirty adult participants (15 women and 15 men), aged 29–65 years (mean age 43.2 ± 8.6 years), with Fitzpatrick skin phototype II, were enrolled. Participants were recruited on the basis of facial erythema considered suitable for vascular 1064-nm Nd:YAG laser treatment. A diagnosis of rosacea was not required. No predefined disease-severity scale was used because eligibility was based on the facial erythema phenotype and suitability for vascular laser treatment rather than on a specific dermatologic diagnosis.

The study employed a prospective, randomized, vehicle-controlled split-face design, with each participant serving as their own control. This approach was intended to reduce the influence of interindividual variability in skin properties, vascular reactivity, and individual responses to laser treatment.

Participants were eligible if they presented with facial erythema suitable for vascular Nd:YAG laser treatment, had no contraindications to laser therapy, and were able to comply with the complete treatment, topical-care, and measurement protocol.

Exclusion criteria included active infection or acute inflammatory skin conditions within the treatment area, contraindications to laser therapy, and the use before or during the study of procedures or topical products that could substantially affect the evaluated skin parameters, particularly stratum corneum hydration, epidermal barrier function, skin erythema, or pigmentation.

All participants completed the study protocol and were included in the final analysis (n = 30).

No formal a priori sample-size calculation was performed. The sample size of 30 participants was selected pragmatically for this exploratory within-participant study. The randomized split-face design was chosen to improve statistical efficiency by allowing each participant to serve as their own control and thereby reducing interindividual variability. The absence of an a priori power calculation is acknowledged as a limitation.

### 2.6. Ethical Considerations and Trial Registration

The study was conducted in accordance with the principles of the Declaration of Helsinki. The study protocol was approved by the appropriate Bioethics Committee (approval No. 227/KBL/OIL/2023; 12 December 2023).

Before enrollment, all participants were informed about the purpose and course of the study, the procedures and instrumental assessments involved, and the potential risks associated with participation. Written informed consent for participation and the scientific use of the collected data was obtained from all participants.

Participant recruitment occurred from 8 January 2024 (first enrolment) to 15 January 2024 (final enrolment), and the study was completed on 25 March 2024. The study was subsequently registered in the ISRCTN registry on 26 August 2026 (ISRCTN69645337); registration was therefore retrospective. The study procedures and measurement endpoints had been established before data collection; however, no publicly time-stamped statistical analysis plan was available before the analyses. This limitation is acknowledged below.

### 2.7. Randomization and Blinding

Allocation of the 7% ectoine formulation and the vehicle to the right or left side of the face was performed using computer-generated randomization.

Both formulations were provided in identical containers labeled with codes A and B, preventing identification of the formulation based on packaging appearance. Participants were not informed which formulation contained ectoine.

Investigators performing the instrumental assessments were also blinded to treatment allocation. The randomization code was revealed only after completion of data collection.

### 2.8. Laser Treatment Protocol

Each participant underwent a series of four treatments at 7-day intervals using a 1064-nm Nd:YAG laser (TimeWalker^®^, Fotona, Ljubljana, Slovenia).

During each session, both sides of the face received an identical laser exposure. Treatments were performed using a PS03X handpiece (Fotona d.o.o., Ljubljana, Slovenia) with a 4-mm spot size. The protocol consisted of two treatment sequences targeting the vascular component:

- Two passes in Versa mode, using a fluence of 40 J/cm^2^ and a pulse duration of 25 ms;
- Two passes in FRAC3 mode, using a fluence of 36 J/cm^2^ and a pulse duration of 1 ms.

Each parameter set was delivered in two complete passes. The sequence of exposure, number of passes, treatment parameters, spot size, and handpiece technique were identical on both sides of the face. The only systematically varied component of the protocol was the topical formulation applied after treatment.

### 2.9. Topical Intervention

The first application of the assigned formulation was performed immediately after completion of the first laser treatment. Thereafter, participants applied the assigned formulations twice daily, in the morning and evening, to the corresponding sides of the face throughout the study period.

On days when subsequent laser treatments were scheduled, the final application was performed on the evening preceding the study visit. Participants were instructed not to apply either study formulation or any other active topical product on the morning of the visit.

Participants were instructed to apply each formulation separately to its assigned side of the face and to avoid transferring the products between sides. The amount applied to each facial side was standardized to approximately a pea-sized amount per application.

Following each laser treatment, participants used only the assigned study formulations and a standardized SPF 50+ photoprotective product. Gentle skin cleansing was permitted.

Throughout the study period, participants were instructed not to use retinoids, hydroxy acids, chemical peels, vitamin C-containing products, or other biologically active topical agents that could influence epidermal barrier function, inflammatory responses, skin erythema, or pigmentation. Additional aesthetic procedures involving the face were also prohibited.

### 2.10. Assessment Time Points and Standardization of Measurement Conditions

Instrumental assessments were performed at three predefined time points:

- T0—before the first Nd:YAG laser treatment;
- T1—before the third treatment, following completion of the first two treatment sessions;
- T2—7 days after the fourth and final treatment.

This schedule enabled assessment of both changes developing during serial treatment and responses persisting after completion of the full treatment protocol.

At each time point, measurements were performed separately on the ECT-treated side, vehicle-treated side, and UIRA. Measurements on the ECT and vehicle sides were obtained from corresponding anatomical locations, with consistent positioning of the measurement areas throughout the study.

Before instrumental assessment, participants underwent acclimatization under controlled environmental conditions. Measurements were performed at a room temperature of 20–22 °C and relative humidity of 50–60%.

The assessment included stratum corneum hydration by corneometry, transepidermal water loss (TEWL), skin surface pH, erythema index, melanin index, standardized imaging, and high-frequency skin ultrasonography.

Each instrumental measurement was performed in triplicate at the same standardized anatomical location, and the arithmetic mean of the three consecutive measurements was used for statistical analysis.

### 2.11. Assessment of Stratum Corneum Hydration

Stratum corneum hydration was assessed by corneometry using instrumentation manufactured by Courage + Khazaka electronic GmbH, Cologne, Germany.

Measurements were performed separately at corresponding sites on the ECT- and vehicle-treated sides at T0, T1, and T2. Corneometry values were used as a quantitative measure of relative stratum corneum hydration and were expressed in arbitrary units (AU).

Equivalent measurements were obtained from the UIRA to assess temporal stability of this parameter.

### 2.12. Assessment of Transepidermal Water Loss

Epidermal barrier function was assessed by measuring transepidermal water loss (TEWL) using instrumentation manufactured by Courage + Khazaka electronic GmbH.

Measurements were obtained separately from corresponding anatomical sites on the ECT- and vehicle-treated sides at T0, T1, and T2. Equivalent measurements were obtained from the UIRA to assess temporal stability.

TEWL was expressed in g/m^2^/h, with lower values indicating lower transepidermal water loss and, in the context of the present measurements, greater preservation of epidermal barrier function.

### 2.13. Assessment of Skin Erythema and Melanin

The erythema index and melanin index were assessed using a reflectance-based method with a Mexameter^®^ (Courage + Khazaka electronic GmbH).

Measurements were performed at corresponding skin sites on the ECT- and vehicle-treated sides at T0, T1, and T2. Equivalent measurements were obtained from the UIRA to assess temporal stability.

The erythema index, expressed in arbitrary units (AU), was used as a quantitative measure of the red component associated with the cutaneous vascular response. The melanin index, also expressed in AU, was used to quantify the pigmentation component and to assess the specificity of observed color changes with respect to vascular rather than pigmentary alterations.

### 2.14. Assessment of Skin Surface pH

Skin surface pH was assessed using a skin-specific pH electrode within the Courage + Khazaka electronic GmbH measurement system.

Measurements were performed separately at corresponding anatomical sites on the ECT- and vehicle-treated sides under controlled environmental conditions at T0, T1, and T2. Equivalent measurements were obtained from the UIRA to assess temporal stability.

Skin surface pH was considered an additional parameter characterizing the cutaneous surface environment throughout the study protocol.

### 2.15. Standardized CIELAB Image Analysis

Standardized facial photographs were acquired under controlled and reproducible imaging conditions, maintaining consistent participant positioning, image geometry, and illumination. Corresponding regions of interest (ROIs) were selected on the ECT- and vehicle-treated sides. ROI location and dimensions were kept constant across consecutive assessments for each participant.

Digital colorimetric image analysis was performed in the Python environment. Pixel values within the predefined ROIs were converted from the RGB color space to the CIELAB (L*a*b*) color space, after which the mean a* value was calculated for each ROI.

The a* coordinate represents the position along the green–red color axis, with higher positive values indicating a greater red component.

For each participant, the change in a* between baseline and the final assessment was calculated separately for both sides:

Δa* = a*final − a*baseline

The intervention effect was subsequently expressed as the difference between within-side changes:

ΔΔa* = Δa*ECT − Δa*vehicle

A more negative Δa* value indicated a greater reduction in the red component.

CIELAB analysis was used as an independent image-based quantitative assessment of changes in skin redness, complementary to the instrumental erythema index obtained using the Mexameter^®,^ Courage + Khazaka Electronics GmbH, Cologne, Germany.

### 2.16. High-Frequency Skin Ultrasonography

High-frequency ultrasonographic assessment was performed using a DRAMIŃSKI DermaMed system equipped with a 48-MHz probe(Dramiński S.A., Sząbruk, Poland).

Ultrasound images were acquired from corresponding skin areas on the ECT- and vehicle-treated sides before initiation and after completion of the study protocol. Equivalent measurements were obtained from the UIRA to assess temporal stability.

Echogenicity analysis was performed within standardized ROIs encompassing the dermis. ROIs were positioned within the appropriate tissue layer, excluding the space above the skin surface and the subcutaneous tissue. Identical anatomical criteria were applied when defining ROI boundaries across all images.

Ultrasound acquisition and ROI definition were performed within the blinded instrumental-assessment workflow. Treatment identity remained concealed during data collection, and identical anatomical and image-analysis criteria were applied to ECT and vehicle images.

The proportion of low-echogenic pixels (LEPs) was used for quantitative echogenicity assessment. Pixels with grayscale intensity values of 0–30 on an 8-bit scale (0–255) were classified as low-echogenic. This fixed threshold was used as an operational definition of very low echogenicity and was applied identically to all images and treatment conditions. It should not be interpreted as a universal biological cutoff, and threshold-dependent LEP values are therefore interpreted comparatively within this standardized image-analysis protocol.

The LEP value was calculated as:

LEP (%) = (number of pixels with grayscale values 0–30/total number of pixels within the ROI) × 100

For each side, the LEP value was determined before initiation and after completion of the protocol. Change was calculated as:

ΔLEP = LEPpost − LEPpre

Negative ΔLEP values therefore indicated a reduction in the proportion of low-echogenic pixels within the dermis.

### 2.17. Study Endpoints

The prespecified primary endpoint for the facial study component was the between-intervention difference in the temporal trajectory of the instrumental erythema index measured with the Mexameter^®^. CIELAB a* derived from standardized digital image analysis was used as an independent confirmatory assessment of the red component and was not treated as a co-primary endpoint.

Secondary and supportive endpoints included stratum corneum hydration, TEWL, melanin index, skin surface pH, CIELAB a*, and the proportion of low-echogenic pixels determined by high-frequency ultrasonography.

This multimodal approach enabled characterization of the cutaneous response at complementary levels, including epidermal barrier function, the vascular-associated red component, pigmentation-related parameters, and ultrasonographic properties of the skin.

### 2.18. Statistical Analysis

Statistical analyses were performed in Python 3.13.5 using the NumPy 2.3.5, pandas 2.2.3, SciPy 1.17.0, and statmodels o.14.6libraries. Given the within-subject nature of the split-face design, measurements obtained from the ECT- and vehicle-treated sides were treated as paired observations. Continuous variables are presented as the mean ± standard deviation (SD). For key between-intervention differences, 95% confidence intervals (95% CIs) were also calculated. For parameters assessed at three time points (T0, T1, and T2)—corneometry, TEWL, erythema index, melanin index, and skin surface pH—a two-way repeated-measures analysis of variance (ANOVA) was performed with two within-subject factors: intervention (ECT vs. vehicle) and time (T0, T1, and T2). The principal effect of interest was the intervention × time interaction, which determined whether the temporal trajectory of each parameter differed between the ECT- and vehicle-treated sides. Effect sizes for ANOVA effects are reported as partial eta squared (ηp^2^).

For planned comparisons, changes between individual time points were calculated separately for the ECT- and vehicle-treated sides as follows:

ΔT0–T1 = T1 − T0

ΔT1–T2 = T2 − T1

ΔT0–T2 = T2 − T0

The within-subject intervention effect was defined as the difference between changes:

ΔΔ = ΔECT − Δvehicle

Planned comparisons of within-subject changes between ECT and vehicle were performed using paired-samples Student’s *t*-tests. Where multiple comparisons were conducted, *p*-values were adjusted using the Holm method. Effect sizes for paired differences are reported as Cohen’s d_z.

For parameters assessed at two time points, namely CIELAB a* and LEPs, the primary unit of analysis was the individual change between the final and baseline assessments. Changes on the ECT- and vehicle-treated sides were compared using a paired-samples Student’s *t*-test, and the intervention effect is presented as ΔΔ with its 95% CI and Cohen’s d_z.

For LEPs, an additional sensitivity analysis was performed using the Wilcoxon signed-rank test for paired samples to assess the robustness of the result to the assumptions underlying the parametric analysis. The proportion of participants demonstrating a greater LEP reduction on the ECT-treated side than on the vehicle-treated side was additionally calculated as a measure of individual directional consistency.

Data obtained from the UIRA were analyzed separately from the primary randomized ECT–vehicle comparison. For parameters assessed at T0, T1, and T2, temporal stability within the UIRA was evaluated using a one-way repeated-measures ANOVA, with time as the within-subject factor. Effect sizes are reported as partial η^2^ (ηp^2^). For parameters assessed only before and after completion of the protocol, individual change from baseline to the final assessment was analyzed.

The UIRA constituted a supportive reference analysis and was not treated as a third study arm. Because of its different anatomical location, no direct inferential comparisons of absolute values were performed between the UIRA and the laser-treated facial areas. The primary efficacy analysis remained the randomized within-subject comparison between ECT and vehicle.

All statistical tests were two-sided, and statistical significance was set at α = 0.05.

## 3. Results

### 3.1. Physicochemical Properties of the Formulations

The physicochemical properties of the tested formulations, including pH, electrical conductivity, water activity, and apparent viscosity, are presented in Table 3. The pH values of the two formulations were comparable: 4.84 ± 0.02 for the vehicle and 4.87 ± 0.02 for ECT. Electrical conductivity was higher for the vehicle than for the 7% ectoine formulation.

The apparent viscosity of both hydrogel formulations decreased with increasing shear rate and temperature. Across the investigated shear-rate range and at all tested temperatures, both formulations exhibited non-Newtonian shear-thinning behavior.

**Table 3:** Physicochemical properties of the tested formulations.

Formulation	pH	T_i_ (°C)	Viscosity, ηapp (Pa·s), 0.1 s^−1^	Viscosity, ηapp (Pa·s), 1 s^−1^	Viscosity, ηapp (Pa·s), 10 s^−1^	Electrical Conductivity (mS/cm)	Water Activity
PLA	4.84 ± 0.02	10	4.380 ± 0.053	3.686 ± 0.018	2.102 ± 0.018	2.157 ± 0.015	0.9927 ± 0.0021
PLA	4.84 ± 0.02	20	3.577 ± 0.002	3.264 ± 0.003	1.918 ± 0.006	2.157 ± 0.015	0.9927 ± 0.0021
PLA	4.84 ± 0.02	32	1.893 ± 0.003	1.866 ± 0.004	1.187 ± 0.001	2.157 ± 0.015	0.9927 ± 0.0021
ECT	4.87 ± 0.02	10	5.591 ± 0.004	4.881 ± 0.006	2.724 ± 0.003	1.963 ± 0.015	0.9795 ± 0.0018
ECT	4.87 ± 0.02	20	5.112 ± 0.002	4.122 ± 0.007	2.439 ± 0.007	1.963 ± 0.015	0.9795 ± 0.0018
ECT	4.87 ± 0.02	32	2.032 ± 0.007	2.020 ± 0.011	1.422 ± 0.010	1.963 ± 0.015	0.9795 ± 0.0018

Water activity, which reflects the availability of free water within a formulation, was high for both hydrogels, with values of 0.9927 for the vehicle and 0.9795 for ECT.

### 3.2. Epidermal Barrier-Related Parameters

#### 3.2.1. Corneometry

A strong intervention × time interaction was observed for corneometry (F(2,58) = 153.07; *p* < 0.001; ηp^2^ = 0.841), indicating markedly different temporal trajectories between the ECT- and vehicle-treated sides.

At T0, corneometry values were nearly identical, with mean values of 30.61 ± 8.16 AU for ECT and 30.75 ± 9.01 AU for the vehicle. Between T0 and T1, corneometry increased on both sides; however, the increase was substantially greater on the vehicle-treated side (+25.26 AU) than on the ECT-treated side (+8.85 AU). The within-subject difference in change was −16.41 AU (95% CI: −19.09 to −13.73; *p*_Holm < 0.001; d_z = −2.28).

Between T1 and T2, the direction of change differed between the two treatment areas. Corneometry continued to increase on the ECT-treated side by +4.55 AU, whereas it decreased by −17.83 AU on the vehicle-treated side. The between-intervention difference in change was +22.38 AU (95% CI: 19.03 to 25.72; *p*_Holm < 0.001; d_z = 2.50).

The underlying participant-level data and calculations were rechecked in response to peer review. The marked vehicle-side increase from T0 to T1 (+25.26 AU) followed by the decrease from T1 to T2 (−17.83 AU) was confirmed and was not attributable to a transcription or group-allocation error.

**Figure 2:**
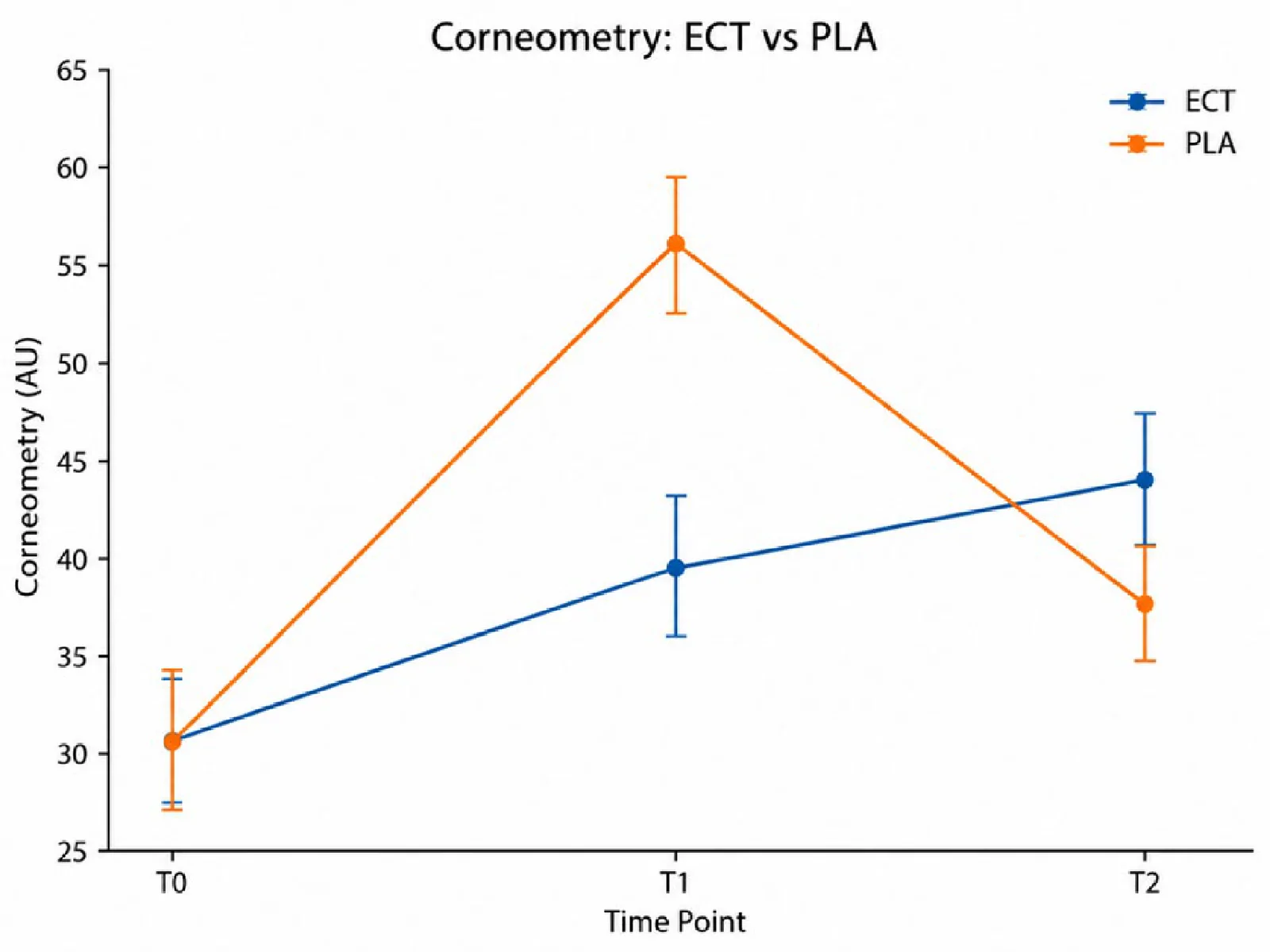
Temporal changes in corneometry on the ECT- and vehicle-treated sides at T0, T1, and T2. Data are presented as mean ± 95% CI.

At T2, mean corneometry was 44.00 ± 7.65 AU for ECT and 38.18 ± 7.92 AU for the vehicle. Across the entire T0–T2 period, corneometry increased by +13.40 AU on the ECT-treated side and +7.43 AU on the vehicle-treated side. The within-subject difference in overall change was +5.96 AU (95% CI: 4.06 to 7.87; *p*_Holm < 0.001; d_z = 1.17). The temporal changes in corneometry are shown in Figure 2.

#### 3.2.2. Transepidermal Water Loss

A significant intervention × time interaction was observed for TEWL (F(2,58) = 5.41; *p* = 0.007; ηp^2^ = 0.157).

At T0, mean TEWL values were 12.24 ± 5.17 g/m^2^/h on the ECT-treated side and 12.88 ± 5.79 g/m^2^/h on the vehicle-treated side. Between T0 and T1, TEWL decreased by −3.32 g/m^2^/h with ECT and −3.50 g/m^2^/h with the vehicle. The difference in change between interventions was small and not statistically significant (ΔΔ = +0.18 g/m^2^/h; 95% CI: −0.59 to 0.94; *p*_Holm = 0.639; d_z = 0.09).

A significant between-intervention difference emerged during the later observation period. Between T1 and T2, TEWL decreased by a further −1.83 g/m^2^/h on the ECT-treated side compared with −0.93 g/m^2^/h on the vehicle-treated side. The within-subject difference in change was −0.90 g/m^2^/h (95% CI: −1.49 to −0.32; *p*_Holm = 0.0075; d_z = −0.58).

**Figure 3:**
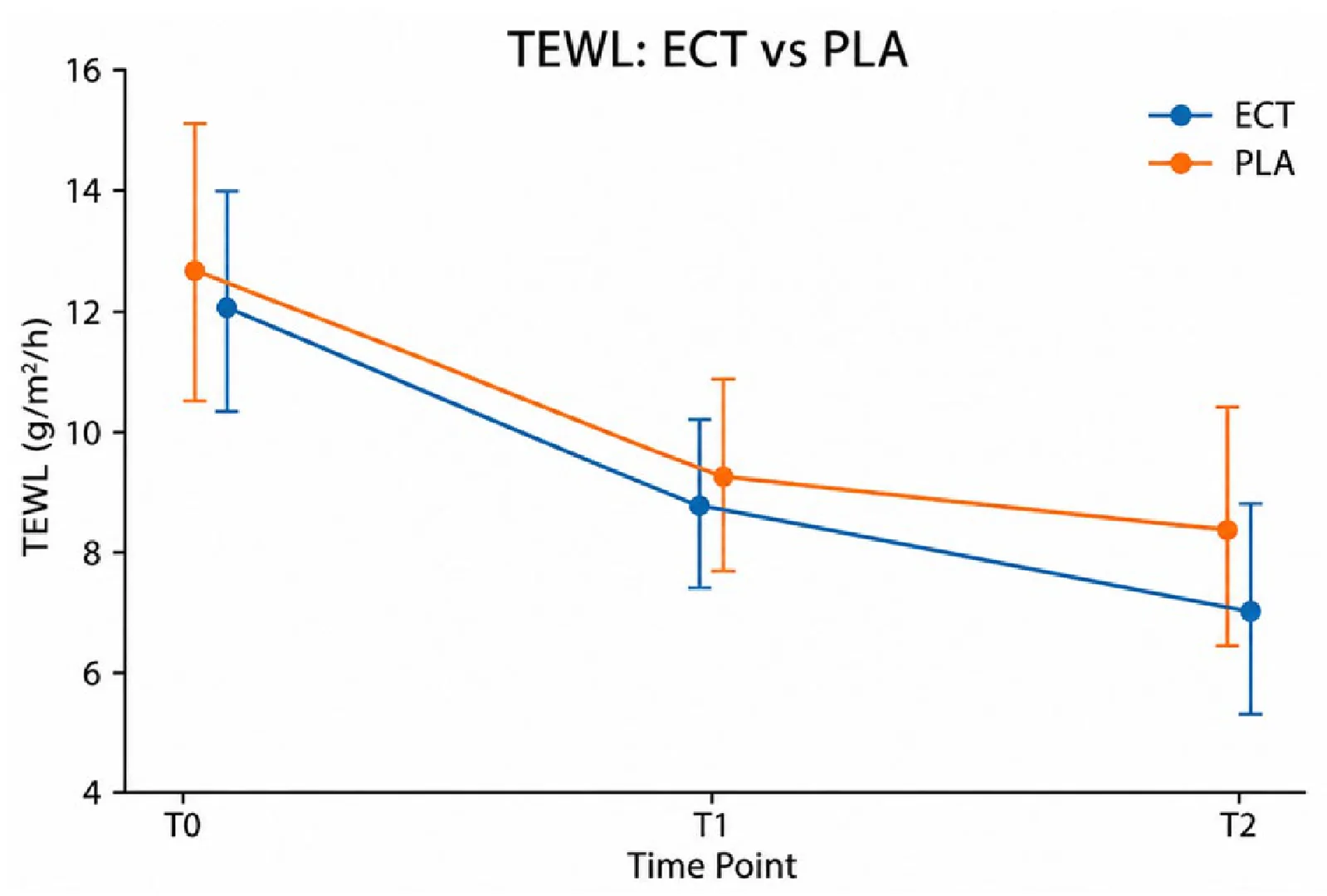
Temporal changes in TEWL on the ECT- and vehicle-treated sides at T0, T1, and T2. Data are presented as mean ± 95% CI.

At T2, mean TEWL was 7.08 ± 4.67 g/m^2^/h for ECT and 8.45 ± 5.17 g/m^2^/h for the vehicle. Across the entire T0–T2 period, TEWL decreased by −5.15 g/m^2^/h on the ECT-treated side and −4.43 g/m^2^/h on the vehicle-treated side. The difference in overall change was −0.73 g/m^2^/h (95% CI: −1.10 to −0.36; *p*_Holm = 0.0012; d_z = −0.73). The complete results for corneometry and TEWL are summarized in Table 4. The temporal changes in TEWL are shown in Figure 3.

**Table 4:** Epidermal Barrier-Related Parameters.

Parameter	Time Point/Change	ECT, Mean ± SD	Vehicle, Mean ± SD	ΔECT − ΔVehicle (95% CI)	*p*_Holm
Corneometry (AU)	T0	30.61 ± 8.16	30.75 ± 9.01	—	—
	T1	39.46 ± 8.20	56.01 ± 9.96	—	—
	T2	44.00 ± 7.65	38.18 ± 7.92	—	—
	ΔT0–T1	+8.85	+25.26	−16.41 (−19.09 to −13.73)	<0.001
	ΔT1–T2	+4.55	−17.83	+22.38 (19.03 to 25.72)	<0.001
	ΔT0–T2	+13.40	+7.43	+5.96 (4.06 to 7.87)	<0.001
TEWL (g/m^2^/h)	T0	12.24 ± 5.17	12.88 ± 5.79	—	—
	T1	8.91 ± 3.82	9.38 ± 4.07	—	—
	T2	7.08 ± 4.67	8.45 ± 5.17	—	—
	ΔT0–T1	−3.32	−3.50	+0.18 (−0.59 to 0.94)	0.639
	ΔT1–T2	−1.83	−0.93	−0.90 (−1.49 to −0.32)	0.0075
	ΔT0–T2	−5.15	−4.43	−0.73 (−1.10 to −0.36)	0.0012

Values at individual time points are presented as mean ± SD. ΔECT − Δvehicle represents the within-subject difference in change between the ECT- and vehicle-treated sides. *p*-values were adjusted using the Holm method.

### 3.3. Erythema and Melanin Indices

#### 3.3.1. Erythema Index

A strong intervention × time interaction was observed for the erythema index (F(2,58) = 27.98; *p* < 0.001; ηp^2^ = 0.491).

Baseline values were comparable, with mean erythema indices of 449.59 ± 88.19 AU on the ECT-treated side and 448.20 ± 89.42 AU on the vehicle-treated side.

A greater reduction in skin erythema was already observed with ECT during the T0–T1 interval. The erythema index decreased by −56.64 AU on the ECT-treated side compared with −38.22 AU on the vehicle-treated side. The within-subject difference in change was −18.42 AU (95% CI: −26.50 to −10.34; *p*_Holm < 0.001; d_z = −0.85).

The greater reduction with ECT persisted during the subsequent observation period. Between T1 and T2, the erythema index decreased by a further −38.35 AU on the ECT-treated side and −21.33 AU on the vehicle-treated side. The difference in change was −17.02 AU (95% CI: −28.31 to −5.73; *p*_Holm = 0.0045; d_z = −0.56).

At T2, the mean erythema index was 354.60 ± 84.07 AU with ECT and 388.65 ± 90.38 AU with the vehicle. Across T0–T2, the erythema index decreased by −94.99 AU with ECT, corresponding to approximately 21.1% of the baseline value, compared with −59.55 AU, or approximately 13.3%, with the vehicle.

The within-subject difference in the overall reduction was −35.44 AU (95% CI: −44.88 to −26.01; *p*_Holm < 0.001; d_z = −1.40), representing a large effect.

**Figure 4:**
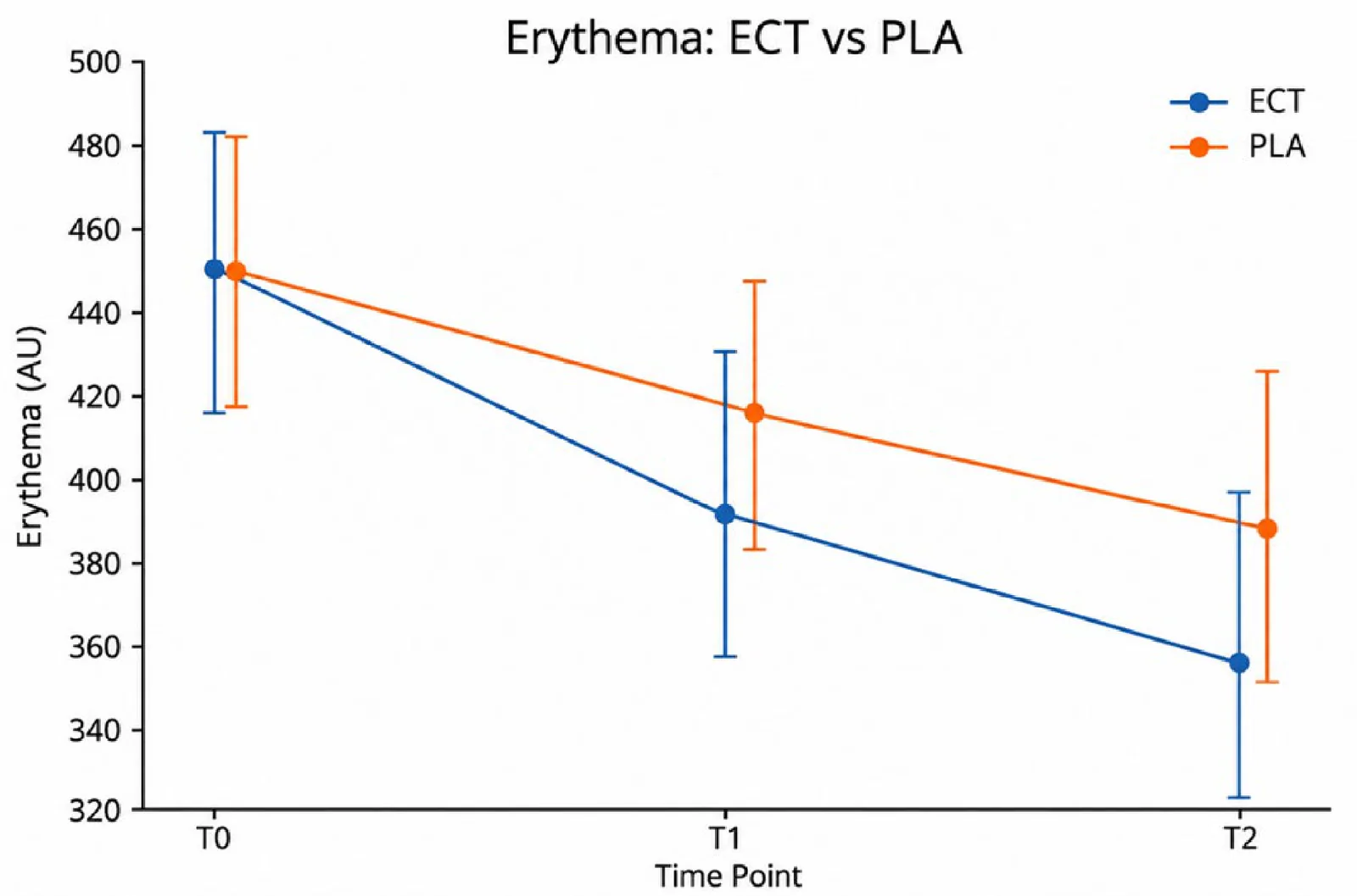
Temporal changes in the erythema index on the ECT- and vehicle-treated sides at T0, T1, and T2. Data are presented as mean ± 95% CI.

Unlike corneometry, for which the direction of the vehicle response changed between T1 and T2, greater reduction in skin erythema with ECT was observed during both consecutive observation intervals. The temporal changes in the erythema index are shown in Figure 4.

#### 3.3.2. Melanin Index

No significant intervention × time interaction was observed for the melanin index (F(2,58) = 0.24; *p* = 0.787; ηp^2^ = 0.008).

Baseline values were 139.51 ± 14.30 AU on the ECT-treated side and 126.50 ± 15.57 AU on the vehicle-treated side. Given this baseline difference, the intervention effect was evaluated on the basis of within-subject changes over time rather than absolute between-side values.

Between T0 and T1, the melanin index changed by −4.00 AU on the ECT-treated side and −1.83 AU on the vehicle-treated side. The difference in change was not significant after Holm adjustment (ΔΔ = −2.18 AU; 95% CI: −4.47 to 0.12; *p*_Holm = 0.187).

Across the entire T0–T2 period, the mean change was +0.47 AU with ECT and +2.42 AU with the vehicle. The within-subject difference in change was −1.95 AU (95% CI: −9.87 to 5.98; *p*_Holm = 1.000; d_z = −0.09). Thus, no significant between-intervention difference in the temporal trajectory of the melanin index was observed. The complete results for the erythema and melanin indices are summarized in Table 5.

**Table 5:** Changes in Erythema and Melanin Indices.

Parameter	Time Point/Change	ECT, Mean ± SD	Vehicle, Mean ± SD	ΔECT − Δvehicle (95% CI)	*p*_Holm
Erythema index (AU)	T0	449.59 ± 88.19	448.20 ± 89.42	—	—
	T1	392.95 ± 94.10	409.98 ± 88.80	—	—
	T2	354.60 ± 84.07	388.65 ± 90.38	—	—
	ΔT0–T1	−56.64	−38.22	−18.42 (−26.50 to −10.34)	<0.001
	ΔT1–T2	−38.35	−21.33	−17.02 (−28.31 to −5.73)	0.0045
	ΔT0–T2	−94.99	−59.55	−35.44 (−44.88 to −26.01)	<0.001
Melanin index (AU)	T0	139.51 ± 14.30	126.50 ± 15.57	—	—
	T1	135.51 ± 12.64	124.67 ± 15.33	—	—
	T2	139.99 ± 23.33	128.92 ± 24.57	—	—
	ΔT0–T1	−4.00	−1.83	−2.18 (−4.47 to 0.12)	0.187
	ΔT1–T2	+4.48	+4.25	+0.23 (−8.79 to 9.25)	1.000
	ΔT0–T2	+0.47	+2.42	−1.95 (−9.87 to 5.98)	1.000

### 3.4. Standardized CIELAB Image Analysis

Colorimetric analysis of the standardized ROIs demonstrated a significant difference in the magnitude of change in the CIELAB a* coordinate between the ECT- and vehicle-treated sides.

Baseline a* values were comparable between treatment areas, with mean values of 15.01 ± 3.48 for ECT and 14.33 ± 3.16 for the vehicle (*p* = 0.161).

At the final assessment, a* decreased on both sides, reaching 11.68 ± 3.13 on the ECT-treated side and 12.19 ± 2.87 on the vehicle-treated side.

The mean change in a* was −3.33 ± 1.21 with ECT and −2.15 ± 1.04 with the vehicle. The within-subject difference in change was −1.18 (95% CI: −1.58 to −0.78; t(29) = −5.99; *p* < 0.001; d_z = −1.09), corresponding to a large effect.

**Figure 5:**
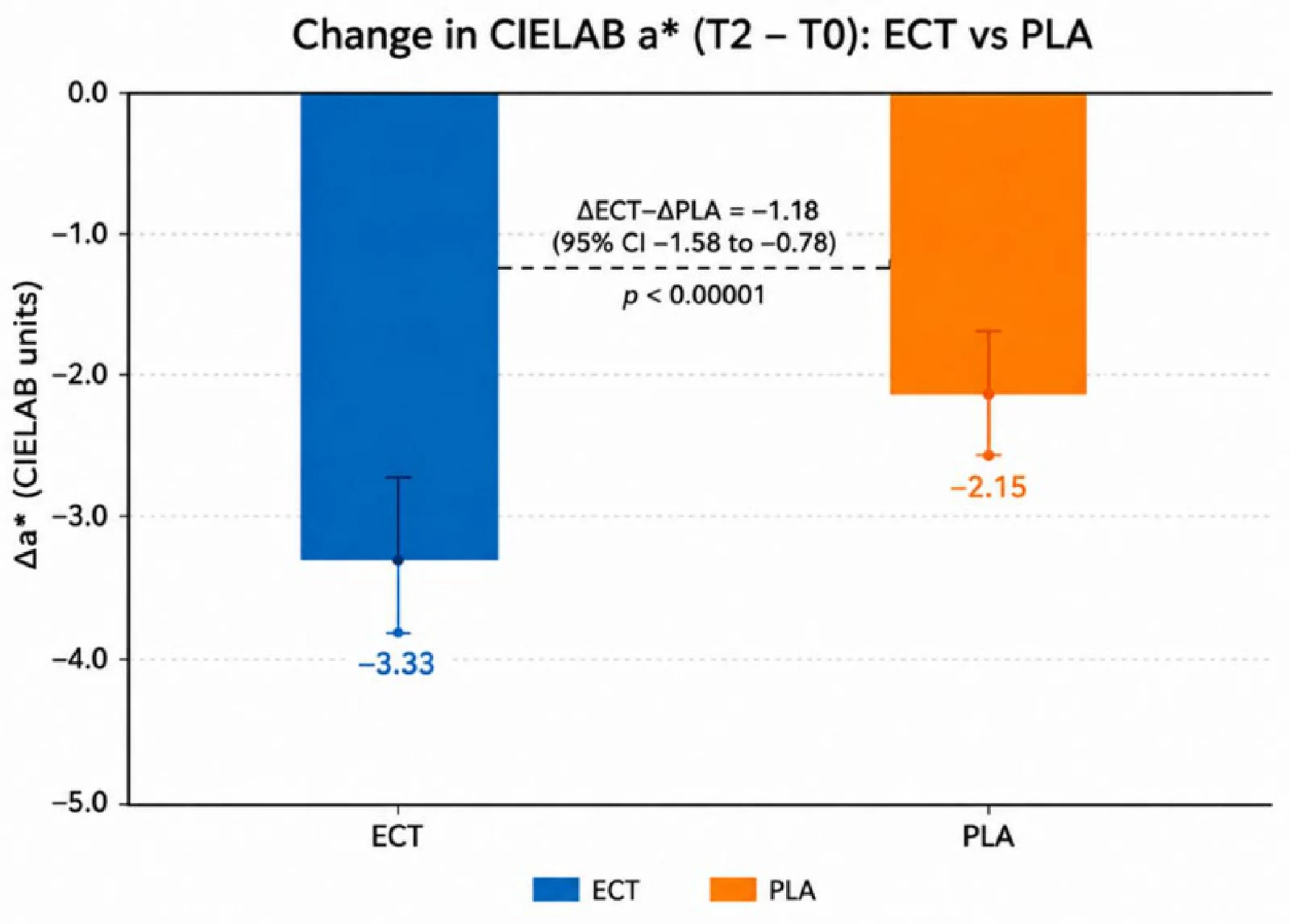
Change in the CIELAB a* coordinate between baseline and final assessment on the ECT- and vehicle-treated sides. Negative Δa* values indicate a reduction in the red component.

A greater reduction in a* on the ECT-treated side than on the vehicle-treated side was observed in 28 of 30 participants (93.3%). The complete CIELAB a results are summarized in Table 6. The change in the CIELAB a coordinate between baseline and final assessment is shown in Figure 5.

**Table 6:** CIELAB a* Colorimetric Analysis.

Parameter	ECT, Mean ± SD	Vehicle, Mean ± SD	ECT − Vehicle/ΔECT − ΔVehicle (95% CI)	*p*
a* at baseline	15.01 ± 3.48	14.33 ± 3.16	+0.68	0.161
a* at final assessment	11.68 ± 3.13	12.19 ± 2.87	−0.50	0.255
Δa*	−3.33 ± 1.21	−2.15 ± 1.04	−1.18 (−1.58 to −0.78)	<0.001

### 3.5. High-Frequency Ultrasonographic Analysis of Low-Echogenic Pixels

High-frequency ultrasonography demonstrated a reduction in the proportion of low-echogenic pixels (LEPs) on both sides of the face, with a significantly greater reduction on the ECT-treated side.

Mean LEPs on the ECT-treated side decreased from 2.92 ± 1.44% at baseline to 1.30 ± 1.32% after completion of the protocol, corresponding to a mean change of −1.61 ± 0.92 percentage points (pp). On the vehicle-treated side, LEPs decreased from 2.48 ± 1.45% to 1.41 ± 1.18%, corresponding to a mean change of −1.07 ± 0.84 pp.

Within-subject comparison demonstrated a significantly greater reduction in LEPs with ECT than with the vehicle. The difference in change was −0.55 pp (95% CI: −0.72 to −0.37; t(29) = −6.38; *p* < 0.001; d_z = −1.16), representing a large effect. The result remained significant in the nonparametric sensitivity analysis (Wilcoxon signed-rank test, *p* < 0.001).

**Figure 6:**
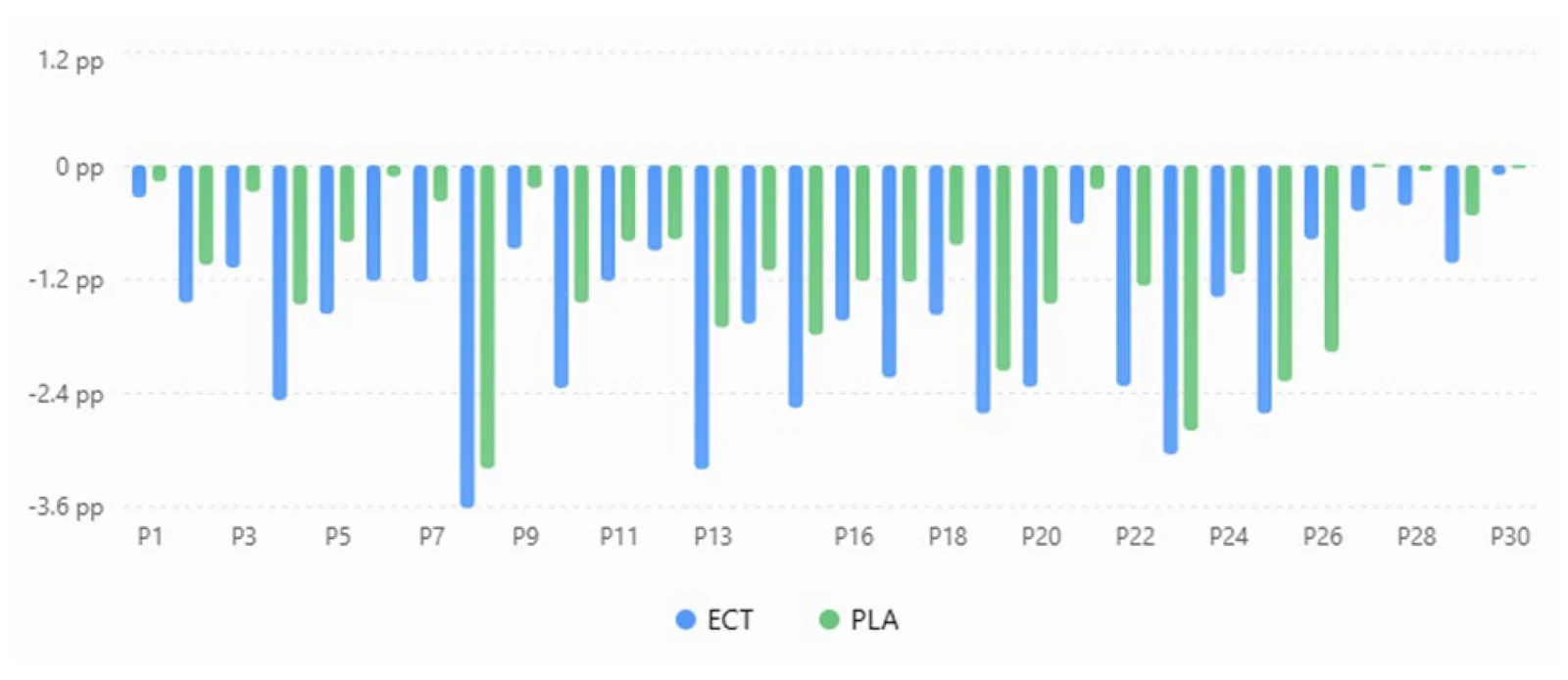
Individual changes in the proportion of low-echogenic pixels (LEPs) between baseline and final assessment on the ECT- and vehicle-treated sides. Negative values indicate a reduction in LEPs. A greater LEP reduction with ECT than with the vehicle was observed in 29 of 30 participants (96.7%).

A greater reduction in LEPs on the ECT-treated side was observed in 29 of 30 participants (96.7%), demonstrating a high degree of within-subject consistency in the direction of the effect. Individual changes in the proportion of low-echogenic pixels (LEPs) are shown in Figure 6. The complete LEP results are summarized in Table 7.

**Table 7:** Changes in the Proportion of Low-Echogenic Pixels (LEPs).

Parameter	ECT, Mean ± SD	Vehicle, Mean ± SD	ΔECT − ΔVehicle (95% CI)	*p*
LEPs at baseline	2.92 ± 1.44%	2.48 ± 1.45%	—	—
LEPs at final assessment	1.30 ± 1.32%	1.41 ± 1.18%	—	—
ΔLEP	−1.61 ± 0.92 pp	−1.07 ± 0.84 pp	−0.55 (−0.72 to −0.37) pp	<0.001

Values are presented as mean ± SD. ΔLEP was calculated as the final value minus the baseline value; negative values indicate a reduction in the proportion of low-echogenic pixels. pp, percentage points.

### 3.6. Changes in Skin Surface pH

Mean baseline skin surface pH values were comparable between the two sides, at 6.07 ± 0.63 for ECT and 6.03 ± 0.51 for the vehicle. At T1, pH increased in both treatment areas to 7.60 ± 2.80 and 7.52 ± 2.71, respectively. At T2, mean values were 6.83 ± 2.15 with ECT and 7.20 ± 2.90 with the vehicle.

Repeated-measures analysis demonstrated a significant effect of time (F(2,58) = 3.45; *p* = 0.039), indicating changes in skin surface pH over the course of the protocol irrespective of the topical formulation. However, no significant advantage of ECT over the vehicle was observed in the temporal trajectory of skin surface pH.

Between T0 and T1, the mean change was +1.53 with ECT and +1.49 with the vehicle, resulting in a minimal between-intervention difference (ΔΔ = +0.04; 95% CI: −0.21 to 0.30; *p* = 0.729; d_z = 0.06).

Across T0–T2, pH changed by +0.76 with ECT and +1.17 with the vehicle. The difference in change was −0.41 (95% CI: −0.97 to 0.15) and was not statistically significant (*p* = 0.146; d_z = −0.27). The complete skin surface pH results are summarized in Table 8.

**Table 8:** Changes in Skin Surface pH.

Time Point/Change	ECT	Vehicle	ΔECT − ΔVehicle (95% CI)	*p*	d_z
T0	6.07 ± 0.63	6.03 ± 0.51	—	—	—
T1	7.60 ± 2.80	7.52 ± 2.71	—	—	—
T2	6.83 ± 2.15	7.20 ± 2.90	—	—	—
ΔT0–T1	+1.53	+1.49	+0.04 (−0.21 to 0.30)	0.729	0.06
ΔT1–T2	−0.77	−0.32	−0.46 (−1.07 to 0.16)	0.141	−0.28
ΔT0–T2	+0.76	+1.17	−0.41 (−0.97 to 0.15)	0.146	−0.27

Effect of time: F(2,58) = 3.45; *p* = 0.039.

### 3.7. Safety and Tolerability

No participant reported an adverse event attributable to either the 7% ectoine formulation or the vehicle during the study, and no participant discontinued treatment because of tolerability concerns. No formal standardized tolerability scale or patient-reported symptom instrument was used; therefore, safety findings are reported descriptively on the basis of participant-reported adverse events.

### 3.8. Summary of the Main Intervention Effects

The main within-subject intervention effects across the assessed instrumental outcomes are summarized in Table 9.

**Table 9:** Summary of the main within-subject intervention effects.

Parameter	Main Comparison	ECT	Vehicle	ΔΔ ECT − Vehicle	95% CI	*p*	d_z
Corneometry (AU)	ΔT0–T2	+13.40	+7.43	+5.96	4.06 to 7.87	<0.001	1.17
TEWL (g/m^2^/h)	ΔT0–T2	−5.15	−4.43	−0.73	−1.10 to −0.36	0.0012	−0.73
Erythema index (AU)	ΔT0–T2	−94.99	−59.55	−35.44	−44.88 to −26.01	<0.001	−1.40
Melanin index (AU)	ΔT0–T2	+0.47	+2.42	−1.95	−9.87 to 5.98	1.000	−0.09
Skin surface pH	ΔT0–T2	+0.76	+1.17	−0.41	−0.97 to 0.15	0.146	−0.27
CIELAB a*	Δbaseline–final	−3.33	−2.15	−1.18	−1.58 to −0.78	<0.001	−1.09

### 3.9. Integrated Analysis of Instrumental Outcomes

Multiparametric analysis demonstrated differences between ECT and the vehicle across epidermal barrier-related parameters, the erythematous component, and ultrasonographic skin characteristics.

Regarding epidermal barrier-related parameters, the ECT-treated side demonstrated a greater overall improvement in corneometry and a greater reduction in TEWL. Corneometry showed a strong intervention × time interaction (ηp^2^ = 0.841), with distinctly different temporal response patterns between the two sides. The vehicle-treated side showed a marked increase at T1 followed by a pronounced decrease at T2, whereas the ECT-treated side showed a more gradual increase that was maintained through the final assessment. For TEWL, the initial reduction was comparable between sides, whereas during the later observation period, the further decrease in transepidermal water loss was greater with ECT.

Particularly pronounced between-intervention differences were observed for parameters reflecting the erythematous component. The Mexameter-derived erythema index decreased from T0 to T2 by 94.99 AU with ECT and 59.55 AU with the vehicle, corresponding to a difference in change of −35.44 AU (*p* < 0.001; d_z = −1.40). This direction of effect was independently confirmed by standardized CIELAB image analysis: a* decreased by −3.33 with ECT and −2.15 with the vehicle, with a difference in change of −1.18 (*p* < 0.001; d_z = −1.09). A greater reduction in a* with ECT was observed in 28 of 30 participants (93.3%).

High-frequency ultrasonography demonstrated a parallel difference in LEPs. Mean LEPs decreased by 1.61 pp with ECT and 1.07 pp with the vehicle, with a within-subject difference in change of −0.55 pp (95% CI: −0.72 to −0.37; *p* < 0.001; d_z = −1.16). A greater LEP reduction with ECT was observed in 29 of 30 participants (96.7%).

No significant between-intervention difference was observed in the temporal trajectory of the melanin index.

Taken together, these findings demonstrate a coherent multiparametric pattern of differences between ECT and the vehicle, comprising a greater final improvement in stratum corneum hydration and reduction in TEWL, a greater reduction in the erythematous component confirmed by two independent quantitative methods, and a greater reduction in the proportion of low-echogenic pixels on high-frequency ultrasonography.

### 3.10. Stability of Skin Parameters Within the Untreated Internal Reference Area

Within the UIRA, which was neither exposed to laser treatment nor treated with either study formulation, the evaluated skin parameters remained stable across the consecutive assessment time points. Repeated-measures analyses demonstrated no significant effect of time for any of the evaluated parameters.

For corneometry, mean values were 38.75 ± 10.99 AU at T0, 38.59 ± 10.86 AU at T1, and 38.81 ± 11.03 AU at T2, with no significant effect of time (F(2,58) = 1.69; *p* = 0.194; ηp^2^ = 0.055).

Similarly, mean TEWL values remained stable at 12.17 ± 1.31 g/m^2^/h at T0, 12.33 ± 1.09 g/m^2^/h at T1, and 12.36 ± 1.31 g/m^2^/h at T2, with no significant effect of time (F(2,58) = 1.10; *p* = 0.339; ηp^2^ = 0.037).

No significant temporal changes were observed in skin color-related parameters. The mean erythema index was 450.70 ± 69.64 AU at T0, 450.53 ± 69.57 AU at T1, and 450.83 ± 69.63 AU at T2 (F(2,58) = 1.58; *p* = 0.216; ηp^2^ = 0.052). Corresponding melanin index values were 59.27 ± 38.09 AU, 59.30 ± 38.00 AU, and 59.47 ± 38.20 AU, respectively (F(2,58) = 1.62; *p* = 0.207; ηp^2^ = 0.053).

Skin surface pH also remained stable, with mean values of 5.793 ± 0.351 at T0, 5.790 ± 0.348 at T1, and 5.793 ± 0.353 at T2 (F(2,58) = 0.36; *p* = 0.699; ηp^2^ = 0.012).

Collectively, these results indicate an absence of significant temporal variability in the evaluated parameters within untreated skin. The stability of the UIRA therefore provides a supportive within-subject reference for interpreting temporal changes observed in the laser-treated areas. Because of its different anatomical location, the UIRA was not used for direct comparisons of absolute values with the ECT- and vehicle-treated areas; the primary efficacy analysis remained the randomized within-subject ECT–vehicle comparison. The temporal stability of the instrumental skin parameters within the UIRA is summarized in Table 10.

**Table 10:** Stability of Instrumental Skin Parameters within the UIRA during the Observation Period.

Parameter	T0	T1	T2	F(2,58)	*p*	ηp^2^
Corneometry (AU)	38.75 ± 10.99	38.59 ± 10.86	38.81 ± 11.03	1.69	0.194	0.055
TEWL (g/m^2^/h)	12.17 ± 1.31	12.33 ± 1.09	12.36 ± 1.31	1.10	0.339	0.037
Erythema index (AU)	450.70 ± 69.64	450.53 ± 69.57	450.83 ± 69.63	1.58	0.216	0.052
Melanin index (AU)	59.27 ± 38.09	59.30 ± 38.00	59.47 ± 38.20	1.62	0.207	0.053
Skin surface pH	5.793 ± 0.351	5.790 ± 0.348	5.793 ± 0.353	0.36	0.699	0.012

Values are presented as mean ± SD. UIRA, Untreated Internal Reference Area; AU, arbitrary units. *p*-values refer to the effect of time in the repeated-measures analysis. The UIRA constituted a supportive reference analysis and was not treated as a third study arm.

## 4. Discussion

In this prospective, randomized, vehicle-controlled split-face study, topical application of a formulation containing 7% ectoine during a series of vascular 1064-nm Nd:YAG laser treatments was associated with a distinct temporal trajectory of the cutaneous response compared with the identical vehicle formulation without ectoine. The most pronounced between-intervention differences involved the erythematous component, epidermal barrier-related parameters, and ultrasonographic skin properties. By contrast, no significant advantage of ECT was observed for the melanin index or skin surface pH. This pattern suggests that the observed effect was not a nonspecific shift across all measured variables but was restricted to specific domains of the skin response to repeated laser exposure.

Particularly relevant was the agreement between the two independent methods used to assess the erythematous component. Between T0 and T2, the erythema index decreased by 94.99 AU on the ECT-treated side and by 59.55 AU on the vehicle-treated side, resulting in a within-subject difference in change of −35.44 AU and a large effect size (d_z = −1.40). Importantly, the greatest reduction with ECT was observed during both the T0–T1 and T1–T2 intervals, indicating that the between-side difference was maintained throughout the treatment protocol rather than emerging only at the final assessment.

This finding was independently supported by CIELAB colorimetric analysis. The a* coordinate, which represents the red–green color axis and is commonly used for objective quantification of skin redness [26,27], decreased by a mean of 3.33 units on the ECT-treated side compared with 2.15 units on the vehicle-treated side. The between-intervention difference in change was −1.18 units, and a greater reduction in a* with ECT was observed in 28 of 30 participants. Agreement between reflectance-based erythema assessment and digital colorimetric analysis strengthens the interpretation of the observed effect by reducing the likelihood that the finding resulted from the characteristics or limitations of a single measurement technique [26,27].

The reduction in the erythematous component on both sides of the face was consistent with the expected effect of vascular laser therapy. The 1064-nm Nd:YAG laser is used for vascular indications, and previous studies, including studies in rosacea, provide relevant mechanistic and clinical context [1,3,5,6,7]. However, a diagnosis of rosacea was not required in the present study; participants were enrolled on the basis of facial erythema suitable for vascular Nd:YAG treatment. Accordingly, the present findings should not be interpreted as evidence of efficacy specifically in rosacea.

In the present study, however, both sides of the face were exposed to an identical laser protocol. Therefore, the difference between ECT and the vehicle cannot be attributed to differences in laser energy delivery, pulse parameters, or the type of physical stimulus. Rather, the findings indicate that regular application of 7% ectoine between successive laser exposures was associated with modification of the cutaneous response to the treatment series.

These findings should not be interpreted as evidence of a direct vasoconstrictive or vessel-specific effect of ectoine. Current understanding of chronic skin erythema indicates that vascular dysregulation does not function as an isolated process. Vascular responses are closely interconnected with innate immune activation, epidermal barrier dysfunction, and neurogenic mechanisms [1,2,3]. In rosacea, pathways involving TLR2–kallikrein 5–LL-37, NF-κB and MAPK signaling, transient receptor potential channels, and mechanisms related to angiogenesis and vascular reactivity have all been implicated [3]. Accordingly, an erythematous phenotype may reflect interactions among vascular, inflammatory, neurogenic, and barrier-related components rather than merely alterations in vessel diameter [1,2,3].

Within this framework, the greater reductions in both the erythema index and CIELAB a* observed with ECT may be interpreted as reflecting a more favorable response of the surrounding tissue to repeated photothermal stress. Ectoine is an extremolyte synthesized by microorganisms adapted to highly stressful environmental conditions. Its biological properties are primarily related to organization of the aqueous environment surrounding macromolecules and stabilization of biological structures under stress [10,11,12,14]. Available evidence indicates that ectoine can protect proteins, membranes, and cellular structures against different forms of physicochemical stress [10,11,12]. In the context of the present study, the relevant mechanism may therefore be preservation of the cellular microenvironment during repeated physical exposure rather than a direct effect on the vascular target itself.

This mechanism may be particularly relevant to serial treatment protocols. In such settings, the skin does not respond to each laser exposure in isolation. Instead, subsequent exposures occur while adaptive and regenerative processes induced by preceding treatments are still ongoing. An intervention that enhances cellular resilience or supports barrier stability could therefore influence how the skin responds to subsequent exposures [10,11,12,15]. This interpretation is indirectly supported by previous observations involving ectoine in post-laser care. Załęska et al. reported that topical ectoine after fractional CO_2_ laser treatment was associated with favorable changes in redness, skin hydration, and TEWL, with the magnitude of the effect depending on ectoine concentration and application regimen [17].

A second domain in which clear differences between ECT and the vehicle were observed was epidermal barrier function. Baseline corneometry values were comparable between the two sides, whereas over T0–T2 stratum corneum hydration increased by 13.40 AU with ECT and by 7.43 AU with the vehicle. The difference in change was 5.96 AU, corresponding to a large effect size (d_z = 1.17).

Of particular interest was not only the final difference but also the temporal pattern of corneometry. The vehicle-treated side showed a pronounced early increase from T0 to T1 (+25.26 AU) followed by a substantial decrease from T1 to T2 (−17.83 AU), whereas the ECT-treated side showed a smaller initial increase followed by a further increase to T2. These values were verified against the underlying data and were not due to transcription or treatment-side allocation errors. The reason for the pronounced nonlinear vehicle-side fluctuation cannot be established from the present study and may reflect short-term hydration dynamics and/or biological and measurement variability. It should therefore be interpreted descriptively rather than as evidence of a specific mechanism.

This temporal pattern is consistent with mechanistic observations reported by Bow et al. [13], who demonstrated that ectoine affects keratin organization within corneocytes and alters the hydration and dehydration kinetics of the stratum corneum. Greater dispersion of keratin structures and characteristics consistent with improved water retention were observed [13]. Such effects may partly explain the more stable corneometric trajectory observed with ECT in the present study.

The TEWL findings provide an additional element supporting this interpretation. From T0 to T2, TEWL decreased by a mean of 5.15 g/m^2^/h with ECT and 4.43 g/m^2^/h with the vehicle, resulting in a between-intervention difference in change of −0.73 g/m^2^/h. Importantly, no significant between-intervention difference was detected during the first observation interval; instead, the difference emerged between T1 and T2.

The TEWL finding requires a cautious clinical interpretation. Although the overall between-intervention difference in change was statistically significant (ΔΔ = −0.73 g/m^2^/h), its absolute magnitude was small. Therefore, this result should be regarded as a supportive instrumental signal of a modest difference in barrier-related response rather than evidence of a clinically important benefit on its own. Its relevance is strengthened primarily by its concordance with the corneometry findings, while the absence of an established minimal clinically important difference in this setting limits clinical interpretation.

TEWL is one of the principal noninvasive parameters used to characterize epidermal permeability barrier function. Higher TEWL generally reflects greater water loss through the skin and impaired barrier function [19,20,21,22,23]. Accordingly, the greater final reduction in TEWL with ECT may be interpreted as indicating a more favorable functional state of the epidermal barrier at the end of the treatment protocol.

The relationship between barrier function and erythematous skin provides relevant biological context. Previous work in rosacea has demonstrated barrier abnormalities involving the stratum corneum, intercellular junctions, and lipid organization [2], but those observations are cited here as mechanistic background rather than as evidence that the present cohort had rosacea. The concomitant differences in TEWL, corneometry, and erythema in the present study should therefore be interpreted within the broader context of barrier–vascular interactions in erythematous skin.

The present results are also consistent with available clinical evidence regarding topical ectoine. In their systematic review, Kauth and Trusova reported beneficial effects of ectoine-containing topical formulations in inflammatory skin diseases associated with barrier impairment, together with favorable tolerability [16]. Hon et al. likewise reported beneficial changes in selected barrier-related parameters following application of an ectoine-containing emollient [18]. Particularly relevant to the current study is the previous investigation by Załęska et al. involving CO_2_ laser-treated skin, in which the effects of ectoine on post-laser skin parameters varied according to concentration and application regimen [17].

The current study extends these observations to a fundamentally different laser–tissue interaction model. Fractional CO_2_ laser exposure produces controlled ablative disruption of the epidermal barrier, whereas in the present 1064-nm Nd:YAG protocol, vascular structures constituted the primary target and there was no comparable ablative disruption of the skin surface. The observation of a favorable ECT–vehicle difference in this non-ablative model suggests that the potential relevance of ectoine in laser procedures may extend beyond supporting the recovery of a mechanically disrupted epidermal barrier [13,16,17,18].

These findings may indicate a broader role for ectoine as a component of peri-procedural skin care aimed at supporting skin homeostasis during repeated controlled physical stress. This possibility requires confirmation in further studies involving other energy-based technologies and different models of laser–tissue interaction.

An important complement to the surface biophysical measurements was the high-frequency ultrasonographic analysis. LEPs decreased by a mean of 1.61 percentage points on the ECT-treated side and by 1.07 percentage points on the vehicle-treated side. The within-subject difference in change was −0.55 percentage points and was statistically significant, with a large standardized paired effect size. However, the absolute between-intervention difference was small and no established minimal clinically important difference exists for LEPs in this setting. Their biological and clinical significance therefore remains uncertain. The most defensible interpretation is that ECT was associated with a small but consistent difference in dermal echogenic properties; the proportion of LEPs is nonspecific and cannot be interpreted as direct evidence of collagen synthesis or extracellular-matrix remodeling.

High-frequency ultrasonography is a noninvasive method that enables quantitative assessment of skin morphology and echogenic properties. Echogenicity parameters have been used to evaluate structural skin characteristics and to monitor responses to dermatological and aesthetic interventions [28,29,30].

However, LEPs require cautious interpretation. The proportion of low-echogenic pixels may reflect aspects of dermal structural organization and tissue water content, but it is not a specific marker of any single extracellular matrix component [28,29,30]. Therefore, the observed reduction in LEPs should not be interpreted as a direct increase in collagen content or as histological evidence of extracellular matrix remodeling.

The most defensible interpretation is that the ECT-treated side exhibited a greater change in dermal echogenic properties than the vehicle-treated side. The concurrent presence of favorable differences in LEPs, TEWL, corneometry, erythema index, and CIELAB a* further indicates that the between-intervention difference was detectable not only in surface parameters but also in quantitative imaging of the dermis.

Not all evaluated parameters differed between ECT and the vehicle. Skin surface pH changed over time, but the difference in total T0–T2 change between interventions did not reach statistical significance. The absence of a between-intervention difference in pH is not inconsistent with the TEWL and corneometry findings, because these parameters characterize different aspects of skin surface physiology and do not necessarily respond in parallel to the same intervention. Differences in the responses of individual functional skin parameters have likewise been reported in studies of ectoine-containing emollients [18].

Similarly, no significant intervention × time interaction was observed for the melanin index. This finding is particularly informative when considered together with the pronounced effects on the erythema index and CIELAB a*. The absence of a corresponding between-intervention effect on pigmentation supports the interpretation that the observed optical changes were predominantly associated with reduction in the red component rather than with a parallel alteration in pigmentation. Parallel assessment of erythema and pigmentation is important when objectively characterizing skin color changes [26,27].

A major feature of the present findings is therefore not any single statistically significant outcome, but rather the consistency of the effect across independent measurement methods and different levels of skin assessment. ECT was associated with a greater reduction in the erythema index, a greater decrease in CIELAB a*, a greater final increase in corneometry, a greater reduction in TEWL, and a greater reduction in LEPs. By contrast, no significant ECT advantage was demonstrated for melanin index or skin surface pH.

This pattern reduces the likelihood that the observed effect represents a nonspecific shift across all instrumental parameters. Moreover, the agreement between Mexameter-derived erythema and CIELAB a* for the red component, together with the complementary behavior of corneometry and TEWL for barrier-related function, provides a form of methodological triangulation across independent measurement techniques [19,20,24,25,26,27]. Collectively, the findings support the hypothesis that regular topical application of 7% ectoine during serial 1064-nm Nd:YAG laser therapy may modify the trajectory of the cutaneous response to repeated photothermal stress, with distinct but complementary effects involving epidermal barrier-related parameters and the erythematous component.

### 4.1. Strengths of the Study

A major strength of this study is its randomized, vehicle-controlled split-face design. Each participant served as their own control, and both sides of the face were exposed to an identical laser protocol. This design reduces the influence of interindividual variability in vascular reactivity, epidermal barrier function, skin characteristics, and individual responses to laser therapy.

The use of an identical vehicle formulation on the control side was also important because it allowed the contribution of the formulation base itself to be distinguished from the additional effect associated with 7% ectoine. Investigators performing instrumental assessments remained blinded to treatment allocation.

Another strength was the multimodal instrumental assessment. The erythematous component was evaluated using two independent quantitative methods, barrier-related function was characterized using both corneometry and TEWL, and high-frequency ultrasonography provided an additional imaging-based tissue parameter. This approach enabled assessment of the cutaneous response at several complementary levels [19,20,24,25,26,27,28,29,30].

Importantly, all evaluated parameters remained stable within the untreated UIRA throughout the observation period. This supports the interpretation that the changes observed in the laser-treated areas were not explained solely by spontaneous temporal variability or environmental factors shared during the study period. The UIRA remained a supportive reference analysis and was not considered a third arm of the randomized ECT–vehicle comparison.

### 4.2. Limitations

The present study has several limitations. The sample size was relatively modest (n = 30), and no formal a priori sample-size calculation was performed; the study should therefore be regarded as exploratory despite the statistical efficiency of the randomized within-participant split-face design. The study included a relatively homogeneous population with Fitzpatrick skin phototype II and evaluated a single standardized 1064-nm Nd:YAG laser protocol, which limits generalizability to other skin phototypes, laser parameters, clinical indications, and treatment settings.

The trial was registered retrospectively on 26 August 2026, after recruitment (8–15 January 2024) and study completion (25 March 2024). Although study procedures and measurement endpoints had been established before data collection, the absence of prospective registration and a publicly time-stamped statistical analysis plan limits independent verification of prespecification and should be considered when interpreting the findings. In addition, the study did not include standardized physician-rated erythema assessment, patient-reported symptoms, treatment satisfaction, or quality-of-life outcomes. Consequently, the clinical meaningfulness of the observed instrumental differences cannot be established directly from the present data.

The follow-up period was limited to the early period after completion of the treatment series and therefore does not establish the long-term persistence of the observed effects. In addition, no histological, biochemical, or molecular endpoints were assessed. Consequently, the proposed mechanisms involving cellular stress responses, oxidative pathways, and inflammatory signaling should be regarded as biologically plausible interpretations rather than mechanisms directly demonstrated by the present study.

Finally, the proportion of LEPs is a nonspecific ultrasonographic parameter and should not be interpreted as a direct surrogate for collagen content or extracellular matrix remodeling.

## 5. Conclusions

In this exploratory randomized split-face study of 30 participants with Fitzpatrick skin phototype II and facial erythema suitable for vascular treatment, topical 7% ectoine used during one specific serial 1064-nm Nd:YAG protocol was associated with a different short-term trajectory of instrumentally assessed cutaneous response compared with the identical vehicle.

The clearest between-intervention difference involved the instrumental erythema index, supported by an independent reduction in CIELAB a*. Differences were also observed in corneometry, TEWL, and LEPs; however, the small absolute between-intervention differences in TEWL and LEPs should be interpreted cautiously and do not, in isolation, establish clinically meaningful benefit.

The LEP findings indicate a measurable difference in dermal echogenic properties but do not constitute direct evidence of extracellular matrix remodeling or increased collagen content. Because no standardized physician-rated erythema assessment or patient-reported outcome was included, the present study demonstrates differences in short-term instrumental endpoints rather than confirmed clinical efficacy.

The absence of corresponding between-intervention effects on the melanin index and skin surface pH further indicates that ectoine did not produce a uniform nonspecific alteration across all measured parameters but showed differential effects in domains related to skin erythema, epidermal barrier function, and ultrasonographic properties.

Taken together, the findings support further investigation of topical ectoine as an adjunct during repeated vascular laser treatment. Confirmation in larger prospectively registered studies, with predefined power calculations, broader skin phototypes and clinical indications, standardized clinical and patient-reported outcomes, and longer follow-up, is required before broader clinical generalization.

## Data Availability

The datasets generated and/or analyzed during the current study are available from the corresponding author upon reasonable request. The data are not publicly available because they contain participant-level information subject to privacy and ethical considerations.
